# Associations Between Stress and Hair Cortisol and Their Relationship to Alcohol Use Among Adolescents and Young Adults: An Epidemiological Cohort Study

**DOI:** 10.1111/adb.70018

**Published:** 2025-02-12

**Authors:** Lena Plettenberg, Anja Kräplin, Catharina Voss, Katja Beesdo‐Baum, Hanna Kische

**Affiliations:** ^1^ Behavioral Epidemiology, Institute of Clinical Psychology and Psychotherapy TUD Dresden University of Technology Dresden Germany; ^2^ Work Group Addictive Behaviors, Risk Analysis and Risk Management, Faculty of Psychology TUD Dresden University of Technology Dresden Germany

**Keywords:** adolescence, alcohol, hair cortisol, stress

## Abstract

The relationship between stress, hair cortisol and alcohol consumption has mostly been investigated among clinical and adult study samples, with inconsistent findings. The present study aimed to examine cross‐sectional and longitudinal associations between chronic stress, hair cortisol and average past‐year alcohol consumption within a population‐based sample of adolescents and young adults. At baseline of the epidemiological cohort study, *N* = 1180 individuals aged 14–21 from Dresden, Germany, were assessed (11/2015–12/2016). A maximum *N* = 1055 were analysed in cross‐sectional analyses and a maximum *N* = 722 in longitudinal analyses (1‐year follow‐up). Multivariate linear regression analyses were conducted to reveal cross‐sectional associations between perceived chronic stress, hair cortisol concentration and average past‐year alcohol consumption in males and females. Longitudinally, weighted linear regression models examined relationships between (a) perceived chronic stress at baseline and altered hair cortisol concentration over 1 year, (b) perceived chronic stress/hair cortisol concentration at baseline and altered average alcohol consumption over 1 year and (c) average past‐year alcohol consumption at baseline and altered stress/hair cortisol concentration over 1 year. Cross‐sectionally, no significant relationships were found between stress, hair cortisol and average past‐year alcohol consumption at baseline. In females, higher baseline perceived chronic stress was associated with an increase in hair cortisol concentration over 1 year, whereas no relationship was found in the cross‐sectional analysis between baseline perceived chronic stress and baseline past‐year average alcohol consumption. When using hair cortisol as a biomarker for stress perception, the focus of future research should be on potential time lags between perceived chronic stress and hair cortisol increase.

## Graphical abstract

The present study aimed to examine cross‐sectional and longitudinal associations between chronic stress, hair cortisol and average past‐year alcohol consumption within a population‐based sample of adolescents and young adults. A maximum *N* = 1055 were analysed in cross‐sectional analyses and a maximum *N* = 722 in longitudinal analyses (1‐year follow‐up). In females, higher baseline perceived chronic stress was associated with an increase in hair cortisol concentration over 1 year, whereas no relationship was found in the cross‐sectional analysis between baseline perceived chronic stress and baseline past‐year average alcohol consumption.

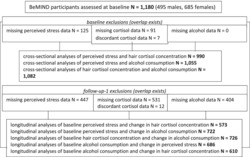

## 1 Introduction

Despite the knowledge that alcohol consumption causes approximately three million deaths worldwide and is involved in more than 133 million disability‐adjusted life years [1], alcohol consumption is still socially accepted and highly traditionalized in many parts of the world. Data of the Federal Center of Health Education revealed that in Germany among young adults between the ages of 18 and 25, almost everyone (94.9%) has tried alcohol at least once. One‐third of them (32.3%) drank alcohol regularly and about two‐fifths (40.6%) practiced binge drinking [2].

To reduce the individual and societal burden of excessive alcohol use, it is important to identify high‐risk individuals at earlier stages. One important risk factor for excessive alcohol use and alcohol use disorders are stressful life events [3]. In particular, the transition from childhood into adolescence has been characterized by a sudden increase of stress, involving significant physical, emotional, social and behavioural changes [4]. To prevent that this stressful period of adolescence increases the risk for alcohol use, it is important to understand the underlying processes linking stress and alcohol use in adolescents.

Cortisol as the hormonal end product of the hypothalamic–pituitary–adrenal (HPA) axis is increasingly of interest as a critical component in the relationship between stress and alcohol consumption in adolescents [5]. Chronic stress leads to changes in the circadian rhythm of cortisol release; hence, the HPA axis becomes more sensitive, releasing a higher amount of cortisol following each stressful event [6]. The relationship between perceived stress and cortisol is complex, with studies showing mixed results. While some findings suggest a positive association, others report no relationship or even negative associations, depending on individual differences, chronicity and context [7, 8]. Chronic stress may lead to HPA axis adaptations, resulting in a blunted cortisol response despite high perceived stress [9]. Perceived stress is also a key predictor of alcohol use, with high levels often linked to greater consumption and binge drinking, supporting the self‐medication hypothesis [10]. Individual factors, such as stress appraisal and coping mechanisms, may modulate this relationship [11, 12]. Understanding these complex interactions between perceived stress, cortisol and alcohol use is critical to identifying mechanisms underlying adolescent alcohol consumption. To gain more insight regarding the relationship between stress, stress responses and alcohol consumption, this paper addresses the need for research in four areas.

The first research need is to investigate the relationship between subjective stress perception and objective markers of the stress response, including their temporal relationship, in order to derive conclusions about the appropriate measurement of stress responses. The review of Staufenbiel et al. [13] reported associations between hair cortisol concentration and subjective stress in less than half of the reviewed 13 studies. Furthermore, Schlotz et al. [14] found stress‐related changes in mood and salivary/plasma cortisol not being synchronized in time. Instead, endocrine responses significantly lagged behind psychological responses.

The second research need relates to how objective and subjective measures of chronic stress differ in their association with alcohol consumption. An association between short‐term cortisol levels and alcohol consumption has been reported [15, 16]. In addition, the analysis of hair cortisol has been established as a promising method to assess long‐term cortisol secretion as a marker of HPA axis activity [17]. Concerning long‐term cortisol secretion, only few studies exist in the field of alcohol use. Stalder et al. [18] showed that adults with alcohol use disorders in acute withdrawal had three to four times higher cortisol concentrations in their hair than abstinent individuals. In accordance to that, Muehlhan et al. [19] found increased hair cortisol concentrations in adult patients with alcohol use disorder compared with controls.

Although this growing body of evidence indicating a relationship between perceived stress and alcohol consumption, as well as between cortisol concentration and alcohol consumption, the temporal sequence of these factors is not yet clear. Studies showing that cortisol interacts with the brain's reward system and promotes habit‐based learning support the notion that increased cortisol concentration may lead to increased alcohol consumption [20]. Some studies support the self‐medication hypothesis, that is, that alcohol use is a strategy for coping with chronic stress among adolescents [21]. In addition, alcohol itself can serve as a stressor [22]. This leads to the third research need, which is to disentangle the temporal relationship between stress, stress responses and alcohol use.

A final research need concerns the study samples. Most studies examining the relationship between stress markers and alcohol consumption have focused on differences between adult individuals with alcohol use disorder and healthy individuals [23, 24]. However, these results cannot be generalized to earlier stages of alcohol use progression, where other processes are known to be involved in explaining the association between stress and alcohol consumption [3]. The authors note that there are inconsistent findings regarding the relationship between a higher frequency of past stressful events and an earlier onset of alcohol consumption, which may be explained by variations in CRF receptor genes that may counteract individual effects when combined. The extent to which moderately elevated alcohol consumption in adolescence is related to stress and the temporal relationship of these factors has been poorly investigated.

Concluding from these four research needs, the present study has two aims: As a primary aim, the cross‐sectional and longitudinal relationships between perceived chronic stress, hair cortisol concentration and alcohol consumption will be examined. As a secondary aim, it will be examined whether perceived chronic stress is related to the cortisol concentration in hair. All analyses will be conducted sex segregated in order to derive indications of sex specificity.

The following hypotheses will be tested:

1. Higher perceived chronic stress is cross‐sectionally associated with higher hair cortisol concentration.
2. Higher perceived chronic stress is longitudinally associated with an increase in hair cortisol concentration over 1 year.
3. Higher hair cortisol concentration and higher perceived chronic stress are both cross‐sectionally associated with higher average past‐year alcohol consumption.
4. Higher hair cortisol concentration and higher perceived chronic stress are both longitudinally associated with an increase in average alcohol consumption over 1 year.
5. Higher average past‐year alcohol consumption is longitudinally associated with an increase in perceived chronic stress and hair cortisol concentration over 1 year.

## 2 Methods

### 2.1 Study Population

The Behaviour and Mind Health (BeMIND) study is a prospective longitudinal epidemiological cohort study investigating a general population sample of 14‐ to 21‐year‐olds from Dresden, Germany. *N* = 6321 individuals were invited to participate after a random age‐ and sex‐stratified sample was drawn from the population register of Dresden in 2015. Due to the criteria of insufficient German language skills, no residence in a Dresden household during the field period and being institutionalized, 893 ineligible participants were excluded. The eligibility of 2708 subjects remained unknown due to non‐response even after two reminder invitation letters. Of the eligible 2720 individuals, 1540 adolescents and young adults were not included due to scheduling conflicts, lack of interest, or other personal constraints. In total, 1180 adolescents and young adults (female: 685 and male: 495) participated in the baseline assessment, corresponding to a response rate of 21.7% (proportion of participants among those identified as eligible plus those with unknown eligibility) and a cooperation rate of 43.4% (proportion of participants among those identified as eligible). Females and those with higher education were more likely to participate. At baseline, a standardized clinical diagnostic interview was conducted with the participants. In the days following, an online questionnaire assessment was administered as well as an Ecological Momentary Assessment. Additional biological/physiological data such as hair cortisol, anthropometric measures, and blood pressure were collected during the final experimental assessment day approximately 1 week after the diagnostic interview. Overall, adolescents and young adults participated for 6–10 h on two assessment days and during 4 days in real life. At baseline, subjects participated in a clinical‐diagnostic assessment (Day 1), in a laboratory assessment approximately 1 week later (Day 2), and in an Ecological Momentary Assessment (EMA) over 4 days in real life and an online questionnaire assessment in between these personal appointments at baseline. The time taken for the standardized interview at baseline varied widely depending on the amount of psychopathology reported and the speed with which the tablet‐based questionnaires were completed (1.5–8 h). If the interviews took longer than 3 h, participants were offered to schedule a separate appointment [25]. Of all baseline participants, 807 (68.2% retention) subjects participated at the smaller‐scale 1 year follow‐up (FU1) with online assessment, interview and biosampling between 02/2017 and 01/2018. The mean time between the baseline assessment and the FU1 was 352.8 days (standard deviation: 66.74). Further details of the study design, procedures, recruitment and sample characteristics were published by Beesdo‐Baum et al. [25]. The ethics committee of the TUD Dresden University of Technology approved the study protocol and its amendments (TUD: EK381102014).

### 2.2 Measures

#### 2.2.1 Alcohol Consumption

Alcohol consumption was assessed within the standardized clinical diagnostic interview, an updated research version (DIA‐X‐5; [26]) of the Munich Composite International Diagnostic Interview (DIA‐X/M‐CIDI; [27]), which was applied face‐to‐face by trained clinical interviewers.

The average alcohol consumption in the past 12 months was calculated using a categorical retrospective quantity‐frequency approach, with a one‐page supplement offering 26 combinations for converting common drinks into standard units per 9 g. This helped respondents specify their typical drinks based on drink categories and serving sizes. Participants were asked if they had at least one glass of alcohol almost every day, three or four times a week, once or twice a week, once to three times a month, or less than once a month in the past 12 months. By multiplying the frequency of alcohol consumption in the past 12 months) with the amount of alcohol consumed per drinking event (standard units), the score gram alcohol per week in the last year was derived. The DIA‐X‐5 showed sufficient test–retest reliability in a convenient sample of adolescents and adults with Cohen's kappa of 0.76 for alcohol consumption and 0.71 for alcohol use disorder as well as sufficient intraclass correlations for time‐related information (age of onset 0.92, age of recency 0.87) [26, 28].

#### 2.2.2 Hair Cortisol

In each subject, two hair strands with a diameter of 3 mm were cut as close as possible to the scalp from the posterior vertex position by trained study personnel. The proximal 3 cm hair were used for analyses, reflecting cortisol secretion during the 3 months preceding the assessment, assuming an average hair growth rate of 1 cm/month. Hair cortisol was measured using a commercially available chemiluminescence immunoassay with high sensitivity (IBL‐International, Hamburg, Germany; [17]). The intraassay and interassay coefficients of variance for this assay are less than 8% [29].

#### 2.2.3 Perceived Chronic Stress

To obtain information regarding chronic stress experiences, the 12‐item Screening Scale of the Trier Inventory for the Assessment of chronic stress (TICS‐SSCS; [30]) was used. Respondents indicated the frequency of stressful situations and experiences within the last 3 months on a 5‐point scale from 0 = *never* to 4 = *very often* [30]. The resulting global scores range from 0 to 48 points, 0 equalling no stress and 48 representing very frequent stress in all five stress domains (chronic preoccupation, work/school‐related and social overload, excessive demands and lack of social recognition). The internal consistency of the TICS‐SSCS can be classified as very good with Cronbach's ɑ = 0.91 [31]. Previously available studies found good external validity in relation to other stress measures and enduring psychological criteria [31]. Internal consistency was 0.89 (Cronbach's alpha).

#### 2.2.4 Covariates

Sex differences in stress levels and cortisol concentrations have been empirically proven by Hampel and Petermann [32], respectively, by Kirschbaum, Wüst and Hellhammer [33], and concerning alcohol consumption by Erol and Karpyak [34]. The older adolescents get, the more stress they experience [35] and the more likely they are to consume alcohol. The computer‐assisted personal interview (DIA‐X‐5) was used to collect information about sex, age, medical history and other sociodemographic details. Several studies indicate age‐dependent changes in HPA axis activity due to the hypothalamic gonadotrophin releasing hormone and thus cortisol secretion during puberty and maturation [36, 37]. Pubertal status was assessed during the online‐questionnaire and the classification of ‘Tanner stages’ was used to provide information concerning pubertal maturation [38]. Self‐ratings on sex‐appropriate schematic drawings were averaged to yield scores from one (prepubertal) to five (adult shape; [39]). Previous research has shown the relationship between increased waist circumference and stress [40], cortisol levels [41] and alcohol consumption [42]. Using a standard tape measure, the waist circumference was taken midway between the lower rib margin and the iliac crest on a horizontal line. A higher frequency of tobacco smoking in stressful situations has been pointed out by Lawless, Harrison, Grandits, Eberly and Allen [43]. Further correlations have been found between smoking and cortisol secretion [44] as well as between smoking and alcohol consumption [45]. Physical activity has been demonstrated to be associated with alcohol consumption [46], as well as with stress [47] and cortisol concentration [21, 48]. The use of oral contraceptives, hair colour, hair treatment with heat and frequency of hair cleansing influence hair cortisol concentrations [49, 50, 51]. Using a brief interview, information concerning physical activity within the past 3 months, smoking habits, the use of oral contraceptives and hair treatment were assessed directly before the hair sample assessment.

### 2.3 Statistical Analysis

First, a descriptive analysis was performed. To ensure that the sample distributions of sex and age were equal to distributions within the target population of 14‐ to 21‐year‐old citizens living in Dresden, sample weights were applied in all quantitative analyses to improve the representativeness of the sample; only N's are reported unweighted. The age and sex distributions of the target population were taken from the Registration Office of the city of Dresden [52] and can be considered highly accurate. The sample was divided into 16 strata according to the 16 possible combinations of sex and age. The sample weights were calculated so that after weighting adjustment, the relative sample frequencies of these groups equal the corresponding relative frequencies of the strata in the population of the 14‐ to 21‐year‐old people of Dresden (the target population). Note that this accounts for (a) intended (sampling probabilities differ across age groups by design) and (b) unintended discrepancies. The distribution of other determinants of participation is also adjusted for to an extent that these are associated with sex and age [25]. A *p*‐value < 0.05 was considered statistically significant. Effects were presented as unstandardized *b*‐coefficients with their 95% confidence interval (CI). The assumptions of the linear regression models were tested by analysing partial regression plots, QQ plots, Wilcoxon rank‐sum test, variance inflation factors (VIF), Breusch–Pagan tests and Durbin Watson tests (see Appendix A).

First, cross‐sectional associations at baseline were examined in age‐, sex‐ and multivariate‐adjusted linear regression models between:

a perceived chronic stress and hair cortisol concentration.
b perceived chronic stress and average past‐year alcohol consumption.
c hair cortisol concentration and average past‐year alcohol consumption.

Second, to examine longitudinal associations, the following age‐, sex‐ and multivariate‐adjusted linear regression models were calculated:

d baseline perceived chronic stress and longitudinal change between hair cortisol concentrations from baseline to FU1.
e baseline hair cortisol concentration and longitudinal change between average past‐year alcohol consumption from baseline to FU1.
f baseline perceived chronic stress and longitudinal change between average past‐year alcohol consumption from baseline to FU1.
g baseline average past‐year alcohol consumption and longitudinal change between perceived chronic stress from baseline to FU1.
h baseline average past‐year alcohol consumption and longitudinal change between hair cortisol concentrations from baseline to FU1.

Parsimonious models were adjusted for sex and age only. Further models were adjusted for sex, waist circumference, smoking status, physical inactivity and age (Multivariate Models 1), respectively, pubertal stage (Multivariate models 2). In analyses with hair cortisol, multivariate models 1 and 2 were additionally adjusted for hair colour, frequency of hair cleaning and hair treatment with heat. Due to literature indications [53], all analyses were additionally conducted separately by sex. The female‐specific multivariate models with hair cortisol concentration were adjusted for the use of oral contraceptives.

The baseline study population comprised 685 females and 495 males (total *N* = 1180). Participants with missing data of the investigated variables (perceived chronic stress, average past‐year alcohol consumption and hair cortisol concentration) as well as discordant values in hair cortisol concentration (more than three standard deviations above or below the mean) were excluded for analyses. The result was a sample size reaching from *n* = 990 to *n* = 1082 for cross‐sectional analyses and *n* = 573 to *n* = 726 for longitudinal analyses. For further details, see Appendix B. All statistical analyses were performed with Stata 14.2 (Stata Corp., College Station, TX, USA) [54].

## 3 Results

Table 1 presents the characteristics of the study population. The mean age was 17.9 years with 48.3% female participants. 76.5% of the population reached high levels of education and 20.7% reported being a current smoker. The average cortisol concentration of the 3 cm hair samples was 7.1 pg/mg. Females had descriptively higher baseline hair cortisol concentrations (7.7 pg/mg) and perceived chronic stress levels (16.9 on a scale of 0–48) than males (6.6 pg/mg; 12.9 on a scale of 0–48). The baseline average weekly alcohol consumption of males (63.9 g/week) was twice that of females (31.8 g/week) in the past year. Within 1 year, the hair cortisol concentration of the total sample decreased by 1.7 pg/mg, with a more pronounced decrease in males (−2.2 pg/mg) than females (−1.3 pg/mg). Perceived chronic stress levels decreased more in females (−3.7) than males (−3.1). While hair cortisol concentration and perceived chronic stress levels decreased in both sexes, average past‐year alcohol consumption only decreased in female participants. While females reduced their weekly alcohol consumption by 12.4 g over 1 year, males increased their alcohol consumption by 3.6 g. A closer look at the age distribution of average past‐year alcohol consumption revealed that up to the age of 16 years for males and up to the age of 17 years for females, more adolescents and young adults increased their alcohol consumption within 1 year than reduced it. From the age of 17 for males and from the age of 18 for females, more adolescents and young adults reduced their average past‐year alcohol consumption than increased it. More details are shown in Table 1. Regarding the variables in Table 1, there was no selective drop‐out from the baseline to FU1 for age, sex, waist circumference, physical inactivity, pubertal stage, hair cortisol and perceived chronic stress. We observed a significant selective drop‐out from baseline to FU1 for education (*χ* ^2^ = 12.96; *p* = 0.005), current smoker (*χ* ^2^ = 8.37; *p* = 0.004), and a significant selective drop‐out for average past‐year alcohol consumption (*z* = 1.99; *p* = 0.046).

**TABLE 1:** Characteristics of the study population.

Baseline characteristics (*n* = 1180)	Weighted	*N*
Age, years, mean	17.9 (0.07)	1180
Sex, %		1180
Female	48.3	685
Male	51.7	495
Education, %		
Low	2.3	25
Middle	18.6	233
High	76.4	881
Other	2.8	41
Waist circumference, cm	78.0 (0.37)	1150
Current smoker, %	20.7	1114
Physically inactive, %	37.0	766
Pubertal stage, %		1045
1	0.1	1
2	0.5	6
3	5.2	70
4	19.1	275
5	75.1	693
Total hair cortisol, pg/mg		
Total sample	7.1 (0.16)	1089
Females	7.7 (0.36)	636
Males	6.6 (0.78)	453
Total alcohol consumption, g/week		
Total sample	48.4 (3.08)	1180
Females	31.8 (2.58)	685
Males	63.9 (5.30)	495
Total perceived stress, scale 0–48		
Total sample	14.9 (0.29)	1055
Females	16.9 (0.39)	626
Males	12.9 (0.42)	429

Developmental characteristics	Weighted	*N*
1‐year change in hair cortisol, pg/mg		
Total sample	−1.7 (0.21)	649
Females	−1.3 (0.40)	394
Males	−2.2 (1.33)	255
1‐year change in alcohol consumption, g/week		
Total sample	−4.4 (3.07)	776
Females	−12.4 (2.94)	462
Males	3.6 (5.37)	314
1‐year change in perceived stress, scale 0–48		
Total sample	−3.4 (0.34)	733
Females	−3.7 (0.45)	444
Males	−3.1 (0.50)	289

*Note:* Data are percentages and mean (SE). *N*s are unweighted.

### 3.1 Cross‐Sectional Associations

Both full‐sample and sex‐specific cross‐sectional analyses between the variables perceived chronic stress and hair cortisol concentration, average past‐year alcohol consumption and perceived chronic stress and hair cortisol concentration and average past‐year alcohol consumption at baseline yielded neither significant results in parsimonious models (see Table 2, for example, cross‐sectional association between perceived chronic stress and hair cortisol concentration: unstandardized *b*‐coefficient: 0.05; 95% CI: −0.06; 0.17) nor in multivariate models (see Appendix C).

**TABLE 2:** Cross‐sectional associations between the variables perceived chronic stress and hair cortisol, hair cortisol and alcohol consumption as well as alcohol consumption and perceived chronic stress at baseline.

Dependent variable
	Independent variable	
*b*‐coefficients (95% CI)	
**Perceived stress**	**Hair cortisol** (*n* = 990)	**Alcohol consumption** (*n* = 1055)
Total sample	0.05 (−0.06; 0.17)	0.00 (−0.01; 0.01)
Males	0.10 (−0.07; 0.27)	0.00 (−0.01; 0.01)
Females	0.06 (−0.10; 0.22)	0.00 (−0.01; 0.01)
**Alcohol consumption**	**Perceived stress** (*n* = 1055)	**Hair cortisol** (*n* = 1082)
Total sample	0.26 (−0.56; 1.07)	0.41 (−0.48; 1.31)
Males	−0.47 (−1.36; 1.45)	0.32 (−1.41; 2.05)
Females	−0.04 (−0.49; 0.56)	0.31 (−0.58; 1.19)
**Hair cortisol**	**Alcohol consumption** (*n* = 1082)	**Perceived stress** (*n* = 990)
Total sample	0.00 (−0.00; 0.01)	0.02 (−0.02; 0.06)
Males	0.00 (−0.00; 0.01)	0.03 (−0.02; 0.09)
Females	0.00 (−0.00; 0.01)	0.02 (−0.03; 0.07)

*Note:* Data are unstandardized *b*‐coefficients and their 95% confidence interval, with *p* < 0.05 marked as *.

### 3.2 Longitudinal Associations

Slightly different results for males compared with females were revealed in sex‐specific longitudinal analyses (see Table 3 for parsimonious models and Appendix C for multivariate models). While the multivariate model 2 in the total sample showed a significant negative association between baseline hair cortisol concentration and 1‐year change in average alcohol consumption (unstandardized *b*‐coefficient: −1.22; 95% CI: −2.43; −0.01), neither the parsimonious nor the multivariate model 1 of the total sample nor the sex‐specific models yielded significant results. In the total sample, average past‐year alcohol consumption at baseline was negatively associated with longitudinal change in perceived chronic stress in multivariate model 1 (unstandardized *b*‐coefficient: −0.01; 95% CI: −0.02; −0.00) and multivariate model 2 (unstandardized *b*‐coefficient: −0.01; 95% CI: −0.02; −0.00), but not in the parsimonious model. Male‐specific models revealed the same significant negative associations in multivariate model 1 (unstandardized *b*‐coefficient: −0.01; 95% CI: −0.02; −0.00) and multivariate model 2 (unstandardized *b*‐coefficient: −0.01; 95% CI: −0.02; −0.00), but not in the parsimonious model (unstandardized *b*‐coefficient: −0.01; 95% CI: −0.02; 0.00). The same models in females did not yield significant results. However, all three female‐specific models showed a significant positive association between baseline perceived chronic stress and 1‐year‐change in hair cortisol concentration (parsimonious model: unstandardized *b*‐coefficient: 0.07; 95% CI: 0.02; 0.12; multivariate model 1: unstandardized *b*‐coefficient: 0.06; 95% CI: 0.01; 0.11; multivariate model 2: unstandardized *b*‐coefficient: 0.07; 95% CI: 0.02; 0.12), whereas such associations were neither found in males nor in the total study sample. Regarding analyses with baseline average past‐year alcohol consumption and 1‐year change in hair cortisol concentration as well as analyses with baseline perceived chronic stress and 1‐year change in average alcohol consumption, significant associations were not found.

**TABLE 3:** Longitudinal associations of perceived chronic stress at baseline with change in hair cortisol over 1 year, perceived chronic stress at baseline with alcohol consumption over 1 year, hair cortisol at baseline with change in alcohol consumption over 1 year, alcohol consumption at baseline with change in perceived chronic stress over 1 year and alcohol consumption at baseline with change in hair cortisol over 1 year.

	Unstandardized *b*‐coefficients (95% CI)	
Dependent variable: change in 1‐year‐FU	Independent variable: baseline	
**1‐year change in hair cortisol**	**Perceived stress** (*n* = 573)	**Alcohol consumption** (*n* = 610)
Total sample	0.04 (−0.01; 0.09)	−0.04 (−0.01; −0.00)
Males	−0.03 (−0.11; 0.05)	−0.00 (−0.01; 0.01)
Females	0.07 (0.02; 0.12)*	−0.00 (−0.01; 0.01)
**1‐year change in alcohol consumption**	**Perceived stress** (*n* = 722)	**Hair cortisol** (*n* = 726)
Total sample	0.37 (−0.69; 1.43)	−0.66 (−1.78; 0.47)
Males	0.60 (−1.75; 2.96)	−1.30 (−3.39; 0.79)
Females	0.19 (−0.42; 0.80)	−0.29 (−1.48; 0.90)
**1‐year change in perceived stress**	**Alcohol consumption** (*n* = 686)	
Total sample	−0.01 (−0.02; 0.00)	
Males	−0.01 (−0.02; 0.00)	
Females	−0.01 (−0.03; 0.02)	

*Note:* Data are unstandardized *b*‐coefficients and their 95% confidence interval, with *p* < 0.05 marked as *.

## 4 Discussion

This paper aimed to examine the cross‐sectional and longitudinal associations between stress, hair cortisol and average past‐year alcohol consumption within a population‐based sample of adolescents and young adults. Because all effect sizes of the significant associations were small, the clinical relevance is questionable. Nevertheless, the results provide evidence for specific mechanisms that contribute to basic research.

### 4.1 Descriptive Data

Adolescents and young adults in the BeMIND sample, with a mean age of 17.9 years, drank an average of 48.4 g alcohol per week. The BeMIND sample follows the typical pattern of alcohol use in adolescence. Males drank more alcohol than females and the older the adolescents are at baseline, the higher the quantities of alcohol they consume [55] (see Appendix D). The number of participants above the cutoff of 10 g/day for females and 20 g/day for males [56] is constantly below 35% and for the younger participants < 17 years of age below 9%. These patterns in the BeMIND study are comparable to reference groups in Germany [55].

In the Netherlands, the proportion of young people who drink alcohol is particularly high at almost 60%. Weekly alcohol consumption among young people is lowest in Lithuania, Cyprus and Latvia, with less than 5% each [57].

However, while the samples' male adolescents increased their weekly alcohol consumption by an average of 3.6 g over the course of a year, females reduced their weekly consumption by an average of 12.4 g. This reduction in weekly alcohol consumption among females aged 14 to 21 years contradicts the general evidence of an increase in alcohol consumption during adolescence and a peak around the age of 21 years for both male sand females [58]. While the decline in our female sample contradicts the general trend of increased consumption during adolescence, it is consistent with findings suggesting greater health awareness among young women [59]. This trend may reflect broader societal changes, including the impact of health promotion campaigns targeting alcohol use (e.g., [60]).

In the BeMIND study, females drank less alcohol but experienced more stress and had higher hair cortisol concentrations compared with males. This is consistent with studies confirming that adolescence girls experience higher levels of stress [61] and consume less alcohol [62] than boys. Approximately 45% of the average 18 years old BeMIND sample have drunk alcohol in the last year, representing a major risk and hence implying the urgency of identifying risk factors in order to implement successful interventions. In this context, stress in adolescence has been identified to be a potential variable in the pathogenesis of risky alcohol consumption [63]. In the following, the results of the present study will be discussed.

### 4.2 Cross‐Sectional Relationship of Hair Cortisol With Stress (Hypothesis 1)

We identified four research needs in the introduction. The fourth research need relates to the study sample of adolescents and young adults. This is addressed in all the discussion sections. The first research need we identified was to investigate the relationship between subjective perceptions of stress and objective markers of stress response. We addressed this research need by testing Hypotheses 1 and 2. Concerning Hypothesis 1, linear regression models revealed no significant associations between baseline stress and hair cortisol concentration. This indicates that among adolescents and young adults, hair cortisol concentration is not suitable as a biomarker to detect long‐term stress of the same timeframe as assessed by the screening scale of the TICS (TICS‐SSCS [31]). While Stalder and Kirschbaum [17] likewise did not find an association between hair cortisol concentration and scores of the TICS‐SSCS, they did find a positive association between hair cortisol concentration and the subscale *social overload* of the complete version of the TICS. It is possible that the items of the screening scale do not represent the corresponding stress dimensions comprehensively enough, thus existing effects did not reach statistical significance. The presented results add to a series of inconclusive findings as gathered in the meta‐analysis of Staufenbiel et al. [13]. Stress is a very complex and multi‐dimensional phenomenon, hence stress from domains not surveyed by the respective questionnaire can distort associations and might explain inconsistent results. For instance, stress due to major life events is often not assessed by psychological stress questionnaires, although positive associations between serious life events and hair cortisol concentrations have been reported [64]. One possible explanation for why hair cortisol might be a better biomarker for serious life events than for chronic stress is that general stress perception is more likely to be distorted by report and recall bias than serious life events [13]. In addition, there are a number of moderators (e.g., coping, resilience and social support) that are probably similarly relevant to daily stress than to severe life events. These moderators reduce distress and might prevent many stressors from leading to chronic stress. Thus, individuals who successfully adapt to many stressors (i.e., high TICS‐SCSS score due to many stressors) might have the same or even lower cortisol levels than individuals who unsuccessfully adapt to a repeated stressor (i.e., low TICS‐SCSS score).

### 4.3 Baseline Stress and Change in Hair Cortisol Over 1 Year (Hypothesis 2)

To disentangle the temporal relationship between subjective perception of stress and objective markers of the stress response, we analysed Hypothesis 2. In the present study, it was only shown among females that hair cortisol rises with a time lag to perceived chronic stress. This links well with Powers et al. [53], stating that the hair cortisol stress response to psychosocial stress in young males is less clear than in young females. One possible reason for this could be that girls become more sensitive to stress during puberty with sex hormones influencing cortisol secretion and the menstrual cycle being associated with diurnal cortisol release [65]. The presented results indicate hair cortisol being suitable as a biomarker for 1‐year past perceived chronic stress in adolescent females. Comparable studies investigating the temporally delayed hair cortisol release in response to experienced stress are not yet available. As the time lag of 1 year is rather long, it remains unclear whether this association would have occurred at another time spot within this year. The question arises as to whether hair cortisol is suitable as a biomarker to measure increased stress in female adolescents and young adults with a time delay, or whether stress must exist over a long period of time for an increased hair cortisol concentration to be detectable.

### 4.4 Cross‐Sectional Relationship of Stress/Hair Cortisol With Average Past‐Year Alcohol Use (Hypothesis 3)

The second research need we identified related to the question of how objective and subjective measures of chronic stress differ in their association with alcohol consumption (Hypothesis 3). Cross‐sectional analyses neither yielded significant associations between baseline hair cortisol concentration and average past‐year alcohol consumption nor between baseline perceived chronic stress and average past‐year alcohol consumption. This links well with findings of Tavolacci et al. [61] showing that among students, perceived chronic stress scores were not associated with general drunkenness and binge drinking. However, in the same sample of Tavolacci, an alcohol use disorder diagnosis in students was positively associated with perceived chronic stress scores. In accordance with that, Watt, Weber, Davies and Forster [63] demonstrated chronic exposure to stress in adolescence being associated with an increased risk of alcohol use disorder.

To our knowledge, no other studies investigated cross‐sectional associations between hair cortisol concentration and average past‐year alcohol consumption in adolescence. Comparable analyses among adult samples by Stalder et al. [18] and Muehlhan et al. [19] demonstrated positive associations between hair cortisol concentration and heavy alcohol consumption.

In summary, previous literature hints at positive associations predominantly between stress/cortisol concentration and problematic alcohol use among adults. The adolescent BeMIND sample, however, is characterized by below‐average alcohol consumption. A possible hypothesis could be that general alcohol consumption rates in adolescence are not related to stress experiences or hair cortisol concentration.

### 4.5 Baseline Stress/Hair Cortisol and Change in Average Alcohol Use Over 1 Year (Hypothesis 4)

The third identified research need was to understand the temporal relationship between stress, stress reactions and alcohol consumption. The longitudinal results showed that baseline stress was not associated with any change in average alcohol consumption over 1 year. This is consistent with findings by Clay, Stafford and Parker [66], who had no evidence of a longitudinal relationship between high stress levels during the COVID‐19 pandemic and increased alcohol consumption among 19‐year‐olds. However, among 30‐year‐olds, stress has been shown to be associated with increased alcohol consumption. Further findings of positive associations between stress and alcohol consumption among adults [67] together with no such associations among adolescents [68] suggest that age might have a moderating effect on the relationship between stress and alcohol use. This suggests that alcohol is mostly not commonly used as a stress coping strategy during adolescence. In this age group, alcohol is often consumed to become integrated into peer groups and to express oneself [69]. Stress‐relief as the basic motivation for drinking alcohol might not apply for adolescents and young adults but for older adults, as supported by the data.

With respect to hair cortisol concentration, the longitudinal results of the BeMIND study showed an association between a lower baseline hair cortisol concentration and the development of higher average alcohol consumption over 1 year. However, this effect was not consistent, occurring only in one of the two multivariate models and neither in the parsimonious model nor in sex‐specific models. The relationship between hair cortisol concentration and longitudinal change in average alcohol consumption have hardly been addressed in previous research.

### 4.6 Baseline Alcohol Use and Change in Stress/Hair Cortisol Over 1 Year (Hypothesis 5)

The third research need (temporal relationship) was also addressed by investigating the inverse relationship between alcohol consumption and changes in stress responses. Longitudinal analyses revealed higher average past‐year alcohol consumption as a predictor for a decrease in stress levels over 1 year in the total sample and among males. However, this effect was also not consistent, occurring in both multivariate models, but not in the parsimonious model. One possible explanation for this inconsistency is that confounders might not have been suitable, resulting in the appearance of a statistical effect in multivariate models which does not exist in reality. Because the effect sizes were small, clinical relevance is questionable.

Some previous studies demonstrated longitudinal relationships between alcohol consumption and reduced stress later the day, respectively, a few days later [70]. These studies support the hypothesis of stress‐reducing effects of alcohol consumption [21]. However, the time lag of 1 year is rather long to assume that reduced stress levels appeared due to the stress‐dampening effect of increased alcohol consumption.

One explanation for higher alcohol consumption being associated with reduced stress levels over 1 year might be that alcohol consumption strengthens social affiliation among adolescents and young adults and therefore reduces social stress. That adolescent males appear to be more susceptible to peer pressure compared with adolescent females was shown by the review of McCoy, Dimler, Samuels and Natsuaki [71].

Regarding hair cortisol concentration, the present data revealed no evidence that average past‐year alcohol consumption is a predictor for changing hair cortisol concentrations over 1 year. This does not support the findings of heavy alcohol consumption being a predictor of a flattening of diurnal cortisol rhythm [72] and lower blood cortisol levels [73] among adolescents.

### 4.7 Strengths and Limitations

The population‐based approach, the large‐scale longitudinal study design and the collection of relevant confounding variables are major strengths of the BeMIND study. Adolescence is of special relevance to the research question, as there are not yet many studies on this area. A common difficulty when investigating the association between subjective stress and hair cortisol concentration is the difference in time frame between questionnaires used to assess perceived chronic stress levels and hair samples used to measure hair cortisol concentration [13]. A highlighted strength of the present study is the TICS‐SSCS being adapted to capture perceived chronic stress over the past 3 months, thus matching the 3 cm hair samples under the assumption of an average hair growth of 1 cm per month.

Despite these strengths of the BeMIND study, some limitations arise. Although the extensive data collection in this study provides a rich data set, it is a limitation that the study was not exclusively designed to examine the specific research question of the current manuscript. Some of the assumptions of the linear regression models were slightly violated. This could have led to biased results. However, linear regression is robust to minor violations of the assumptions. Given the large sample size of the study and the coherent results of the analyses, we consider the results to be reliable. The sample was characterized by a high educational level, which restricts the generalization to adolescents and young adults with lower education level. Due to the decision for a rather parsimonious modelling also in the multivariate models, other, possibly relevant, sources of influence such as affective or endocrine‐related disorders were not considered. As with all retrospective self‐reports, recall‐ and reporting bias may have distorted stress experiences and alcohol consumption. Specifically, the categorical approach to assess alcohol consumption does not capture the exact number of drinks consumed. While combining frequency and quantity estimates provides a useful approximation, this method may introduce recall and reporting biases. Future research should consider using diary methods to enhance accuracy in alcohol consumption measurements. Furthermore, the period of average past‐year alcohol consumption did not match the 3 months covered by the TICS‐SSCS to assess perceived chronic stress or the 3 months of hair cortisol concentration. Regarding longitudinal results, it has to be kept in mind, that we observed a selective drop‐out from baseline to FU for education, smoking and alcohol consumption.

### 4.8 Conclusion and Implication

The BeMIND data indicates that hair cortisol may not be suitable as a biomarker for perceived chronic stress. Among females, higher baseline perceived chronic stress was associated with an increase in hair cortisol concentration over 1 year. In the absence of information on whether the stress remained high within this year, the question arises whether hair cortisol is suitable as a biomarker to survey stress in women with a time lag or whether hair cortisol is suitable as a biomarker to detect long‐term stress of more than 1 year. Further longitudinal studies with several follow‐ups should examine different durations and time intervals of stress and hair cortisol. In the BeMIND study, females drank less alcohol but experienced more stress and had higher hair cortisol concentrations compared with males. While females predominantly reduced their alcohol consumption over 1 year, males mostly increased it. This trend of reducing alcohol intake at an early age should be supported by interventions and incentive systems. Given the bidirectional relationships between stress and average past‐year alcohol consumption, it appears reasonable to address stress and alcohol problems together as early as possible in prevention programmes. In order to appropriately select those who might benefit most from such a prevention programme, further large‐scale longitudinal studies of adolescent samples as well as appropriate randomized‐intervention studies (e.g., random assignment to a stress reduction programme) are required.

## Author Contributions

K.B.B. was responsible for the BeMIND study concept and design. K.B.B., H.K. and C.V. contributed to the acquisition of the data, the methodology and the project administration. L.P. performed the analyses, interpreted the findings and drafted the manuscript. H.K., K.B.B. and A.K. provided critical revision of the manuscript for important intellectual content. All authors critically reviewed content and approved final version for publication.

## Ethics Statement

The authors assert that all procedures contributing to this work comply with the ethical standards of the relevant national and institutional committees on human experimentation and with the Helsinki Declaration of 1975, as revised in 2013. Ethics Committee of TUD Dresden University of Technology 381102014.

## Consent

All participants have signed informed consent. In the case of underage participants, parents provided consent.

## Conflicts of Interest

The authors declare no conflicts of interest.

## Supporting information


**Figure S1.** Flow chart of the study population used for analysis. BeMIND, Behaviour and Mind Health Study.
**Table S1.** Cross‐sectional associations between perceived stress and hair cortisol concentration, perceived chronic stress and alcohol consumption and hair cortisol concentration and alcohol consumption at Baseline.
**Table S2.** Longitudinal associations of perceived chronic stress with change in hair cortisol concentration and alcohol consumption over 1 year, hair cortisol concentration with change in alcohol consumption over 1 year, as well as alcohol consumption with change in perceived chronic stress and hair cortisol concentration over 1 year.
**Table S3.** Sex‐specific cross‐sectional associations between perceived chronic stress and hair cortisol concentration, perceived chronic stress and alcohol consumption and hair cortisol concentration and alcohol consumption at baseline.
**Table S4.** Sex‐specific longitudinal associations of perceived chronic stress with change in hair cortisol concentration and alcohol consumption over 1 year, hair cortisol concentration with change in alcohol consumption over 1 year, as well as alcohol consumption with change in perceived chronic stress and hair cortisol concentration over 1 year.
**Table S5.** Alcohol consumption per week and risky drinking in males and females.

## Data Availability

Strict data sharing rules apply to the BeMIND study to ensure privacy/data protection in this still ongoing cohort study with collection of sensitive health data. However, anonymized data used for this publication and the analytical code are shared with interested researchers upon reasonable request to the study PI.
