# Supplementary material for: Associations Between Stress and Hair Cortisol and Their Relationship to Alcohol Use Among Adolescents and Young Adults: An Epidemiological Cohort Study
Source: Addict Biol. 2025 Feb 12;30(2):e70018. doi: 10.1111/adb.70018 (PMC11821724; doi:10.1111/adb.70018)
Supplement: Supplementary file 1 — Figure S1. Flow chart of the study population used for analysis. BeMIND, Behaviour and Mind Health Study. Table S1. Cross‐sectional associations between perceived stress and hair cortisol concentration, perceived chronic stress and alcohol consumption and hair cortisol concentration and alcohol consumption at Baseline. Table S2. Longitudinal associations of perceived chronic stress with change in hair cortisol concentration and alcohol consumption over 1 year, hair cortisol concentration with change in alcohol consumption over 1 year, as well as alcohol consumption with change in perceived chronic stress and hair cortisol concentration over 1 year. Table S3. Sex‐specific cross‐sectional associations between perceived chronic stress and hair cortisol concentration, perceived chronic stress and alcohol consumption and hair cortisol concentration and alcohol consumption at baseline. Table S4. Sex‐specific longitudinal associations of perceived chronic stress with change in hair cortisol concentration and alcohol consumption over 1 year, hair cortisol concentration with change in alcohol consumption over 1 year, as well as alcohol consumption with change in perceived chronic stress and hair cortisol concentration over 1 year. Table S5. Alcohol consumption per week and risky drinking in males and females. [file ADB-30-e70018-s001.docx]

**Index of the Appendix**

*Index of the appendix*

|  |  | Page |
| --- | --- | --- |
| Appendix A | *Assumptions of the linear regression models* | **2** |
| Appendix B | *Flowchart of the study population* | **3** |
| Appendix C | *Detailed result tables of the linear regression models* | **4** |
| Appendix D | *Alcohol consumption per week and risky drinking in males and females* | **8** |

**Appendix A: Assumptions of the linear regression models**

With values between 0.24 and 2.05, the Durbin-Watson statistics indicated the presence of autocorrelations in some analyses, especially in gender-specific models. The range of VIF values of the predictors was between 1.00 and 1.40, indicating an independence of predictors. Breusch-Pagan tests detected in several cases heteroskedasticity. Inspection of the QQ plots revealed that in some linear regression models the residuals were not normally distributed. The partial regression plots showed a number of ambiguous linear relationships between dependent and independent variables.

**Appendix B: Flow chart – Composition of analysis sample**

BeMIND participants assessed at baseline **N = 1,180** (495 males, 685 females)

*baseline exclusions (overlap exists)*

missing cortisol data N = 91

discordant cortisol data N = 7

missing perceived stress data N = 125

missing alcohol data N = 0

cross-sectional analyses of perceived stress and hair cortisol concentration **N = 990**

cross-sectional analyses of perceived stress and alcohol consumption **N = 1,055**

cross-sectional analyses of hair cortisol concentration and alcohol consumption **N = 1,082**

*follow-up-1 exclusions (overlap exists)*

missing perceived stress data N = 447

missing alcohol data N = 404

missing cortisol data N = 531

discordant cortisol data N = 12

longitudinal analyses of baseline perceived stress and change in hair cortisol concentration **N = 573**

longitudinal analyses of baseline perceived stress and change in alcohol consumption **N = 722**

longitudinal analyses of baseline hair cortisol concentration and change in alcohol consumption **N = 726**

longitudinal analyses of baseline alcohol consumption and change in perceived stress **N = 686**

longitudinal analyses of baseline alcohol consumption and change in hair cortisol concentration **N = 610**

*Fig. 1. Flow chart of the study population used for analysis. BeMIND, Behavior and Mind Health Study.*

**Appendix C: Detailed Result Tables**

| **Table 1**  *Cross-sectional associations between perceived stress and hair cortisol concentration, perceived chronic stress and alcohol consumption and hair cortisol concentration and alcohol consumption at Baseline* | | | |
| --- | --- | --- | --- |
| Dependent Variable | Independent Variable | Dependent Variable | Independent Variable |
|  | b-coefficients (95% CI) |  | b-coefficients (95% CI) |
| **perceived stress**  parsimonious model  multivariate model 1  multivariate model 2 | **hair cortisol**  0.05 (-0.06; 0.17)  0.04 (-0.07; 0.15)  0.04 (-0.07; 0.15) | **hair cortisol**  parsimonious model  multivariate model 1  multivariate model 2 | **perceived stress**  0.02 (-0.02; 0.06)  0.01 (-0.02; 0.05)  0.01 (-0.03; 0.05) |
| **alcohol consumption**  parsimonious model  multivariate model 1  multivariate model 2 | **perceived stress**  0.26 (-0.56; 1.07)  0.20 (-0.57; 0.97)  0.18 (-0.63; 0.99) | **perceived stress**  parsimonious model  multivariate model 1  multivariate model 2 | **alcohol consumption**  0.00 (-0.01; 0.01)  0.00 (-0.01; 0.01)  0.00 (-0.01; 0.01) |
| **hair cortisol**  parsimonious model  multivariate model 1  multivariate model 2 | **alcohol consumption**  0.00 (-0.00; 0.01)  0.00 (-0.00; 0.01)  0.00 (-0.00; 0.01) | **alcohol consumption**  parsimonious model  multivariate model 1  multivariate model 2 | **hair cortisol**  0.41 (-0.48; 1.31)  0.68 (-0.25; 1.61)  0.91 (-0.08; 1.90) |
| Data are unstandardized b-coefficients and their 95% confidence interval, with p < 0.05 marked as *. The parsimonious models were adjusted for age and sex only. The multivariate models 1 were adjusted for age, sex, waist circumference, smoking status, and physical inactivity. The multivariate models 2 were adjusted for the same covariates as the multivariate models 1, but for tanner stage instead of age. Analyses with hair cortisol were above that adjusted for hair color, frequency of hair cleaning and hair treatment with heat. | | | |

| **Table 2**  *Longitudinal associations* *of perceived chronic stress with change in hair cortisol concentration and alcohol consumption over one year, hair cortisol concentration with change in alcohol consumption over one year, as well as alcohol consumption with change in perceived chronic stress and hair cortisol concentration over one year* | | |
| --- | --- | --- |
|  | b-coefficients (95% CI) | |
| Dependent Variable: change in 1-year-FU | Independent Variable: Baseline | |
| **1-year change in hair cortisol**  parsimonious model  multivariate model 1  multivariate model 2 | **perceived stress**  0.04 (-0.01; 0.09)  0.04 (-0.01; 0.09)  0.04 (-0.01; 0.10) | **alcohol consumption**  -0.04 (-0.01; -0.00)  -0.00 (-0.01; 0.00)  -0.00 (-0.01; 0.00) |
| **1-year change in alcohol consumption**  parsimonious model  multivariate model 1  multivariate model 2 | **perceived stress**  0.37 (-0.69; 1.43)  0.32 (-0.73; 1.36)  0.32 (-0.76 1.40) | **hair cortisol**  -0.66 (-1.78; 0.47)  -1.04 (-2.22; 0.14)  -1.22 (-2.43; -0.01)* |
| **1-year change in perceived stress**  parsimonious model  multivariate model 1  multivariate model 2 | **alcohol consumption**  -0.01 (-0.02; 0.00)  -0.01 (-0.02; -0.00)*  -0.01 (-0.02; -0.00)* |  |
| Data are unstandardized b-coefficients and their 95% confidence interval, with p < 0.05 marked as *. The parsimonious models were adjusted for age and only. The multivariate models 1 were adjusted for age, sex, waist circumference, smoking status, and physical inactivity. The multivariate models 2 were adjusted for the same covariates as the multivariate models 1, but for tanner stage instead of age. Analyses with hair cortisol were above that adjusted for hair color, frequency of hair cleaning and hair treatment with heat. | | |

| **Table 3**  *Sex-specific cross-sectional associations between perceived chronic stress and hair cortisol concentration, perceived chronic stress and alcohol consumption and hair cortisol concentration and alcohol consumption at baseline* | | |
| --- | --- | --- |
|  | b-coefficients (95% CI) | |
| Dependent Variable | Independent Variable | |
|  | Males | Females |
| **perceived stress** | **hair cortisol** | |
| parsimonious model  multivariate model 1  multivariate model 2 | 0.10 (-0.07; 0.27)  0.05 (-0.11; 0.21)  0.04 (-0.12; 0.20) | 0.06 (-0.10; 0.21)  0.08 (-0.08; 0.23)  0.08 (-0.08; 0.24) |
| **hair cortisol** | **perceived stress** | |
| parsimonious model  multivariate model 1  multivariate model 2 | 0.03 (-0.02; 0.09)  0.02 (-0.04; 0.07)  0.01 (-0.04; 0.07) | 0.02 (-0.03; 0.07)  0.02 (-0.02; 0.07)  0.02 (-0.02; 0.07) |
| **alcohol consumption** | **perceived stress** | |
| parsimonious model  multivariate model 1  multivariate model 2 | -0.47 (-1.36; 1.45)  0.07 (-1.11; 1.25)  -0.00 (-1.26; 1.26) | -0.04 (-0.49; 0.56)  -0.07 (-0.55; 0.40)  -0.09 (-0.60; 0.41) |
| **perceived stress** | **alcohol consumption** | |
| parsimonious model  multivariate model 1  multivariate model 2 | 0.00 (-0.01; 0.01)  0.00 (-0.01; 0.01)  -0.00 (-0.01; 0.01) | 0.00 (-0.01; 0.01)  -0.00 (-0.01; 0.01)  -0.00 (-0.01; 0.01) |
| **alcohol consumption** | **hair cortisol** | |
| parsimonious model  multivariate model 1  multivariate model 2 | 0.32 (-1.41; 2.05)  0.30 (-1.54; 2.14)  0.75 (-1.25; 2.74) | 0.31 (-0.58; 1.19)  0.53 (-0.35; 1.41)  0.65 (-0.28; 1.58) |
| **hair cortisol** | **alcohol consumption** | |
| parsimonious model  multivariate model 1  multivariate model 2 | 0.00 (-0.00; 0.01)  0.00 (-0.00; 0.01)  0.00 (-0.00; 0.01) | 0.00 (-0.00; 0.01)  0.00 (-0.00; 0.01)  0.00 (-0.00; 0.01) |
| Data are unstandardized b-coefficients and their 95% confidence interval, with p < 0.05 marked as *. The parsimonious models were adjusted for age only. The multivariate models 1 were adjusted for age, waist circumference, smoking status, and physical inactivity. The multivariate models 2 were adjusted for the same covariates as the multivariate models 1, but for tanner stage instead of age. Analyses with hair cortisol were above that adjusted for hair color, frequency of hair cleaning and hair treatment with heat. Female-specific models were additionally adjusted for the use of oral contraceptives. | | |

| **Table 4**  *Sex-specific longitudinal associations* *of perceived chronic stress with change in hair cortisol concentration and alcohol consumption over one year, hair cortisol concentration with change in alcohol consumption over one year, as well as alcohol consumption with change in perceived chronic stress and hair cortisol concentration over one year* | | | | |
| --- | --- | --- | --- | --- |
| Dependent Variable | Independent Variable | | | |
| Males | | b-coefficients (95% CI) | | |
| **1-year change in hair cortisol**  parsimonious model  multivariate model 1  multivariate model 2 | | | **Bl perceived stress**  -0.03 (-0.11; 0.05)  -0.02 (-0.11; 0.07)  -0.02 (-0.11; 0.07) | **Bl alcohol consumption**  -0.00 (-0.01; 0.01)  -0.00 (-0.01; 0.01)  -0.00 (-0.01; 0.01) |
| **1-year change in alcohol consumption**  parsimonious model  multivariate model 1  multivariate model 2 | | | **Bl perceived stress**  0.60 (-1.75; 2.96)  0.52 (-1.79; 2.83)  0.46 (-1.92; 2.84) | **Bl hair cortisol**  -1.30 (-3.39; -0.79)  -1.50 (-3.70; 0.71)  -1.80 (-4.12; 0.53) |
| **1-year change in perceived stress**  parsimonious model  multivariate model 1  multivariate model 2 | | | **Bl alcohol consumption**  -0.01 (-0.02; 0.00)  -0.01 (-0.02; -0.00)*  -0.01 (-0.02; -0.00)* |  |
| Females | | |  |  |
| **1-year change in hair cortisol**  parsimonious model  multivariate model 1  multivariate model 2 | | | **Bl perceived stress**  0.07 (0.02; 0.12)*  0.06 (0.01; 0.11)*  0.07 (0.02; 0.12)* | **Bl alcohol consumption**  -0.00 (-0.01; 0.01)  -0.00 (-0.01; 0.01)  -0.01 (-0.02; 0.00) |
| **1-year change in alcohol consumption**  parsimonious model  multivariate model 1  multivariate model 2 | | | **Bl perceived stress**  0.19 (-0.42; 0.80)  0.16 (-0.44; 0.76)  0.21 (-0.41; 0.84) | **Bl hair cortisol**  -0.29 (-1.48; 0.90)  -0.54 (-1.69; 0.62)  -0.69 (-1.90; 0.52) |
| **1-year change in perceived stress**  parsimonious model  multivariate model 1  multivariate model 2 | | | **Bl alcohol consumption**  -0.01 (-0.03; 0.02)  -0.01 (-0.03; 0.01)  -0.01 (-0.03; 0.01) |  |
| Data are unstandardized b-coefficients and their 95% confidence interval, with p < 0.05 marked as *. The parsimonious models were adjusted for age only. The multivariate models 1 were adjusted for age, waist circumference, smoking status, and physical inactivity. The multivariate models 2 were adjusted for the same covariates as the multivariate models 1, but for tanner stage instead of age. Analyses with hair cortisol were above that adjusted for hair color, frequency of hair cleaning and hair treatment with heat. Female-specific models were additionally adjusted for the use of oral contraceptives. | | | | |

**Appendix D: Alcohol consumption per week and risky drinking in males and females**

**Table 5**

*Alcohol consumption per week and risky drinking in males and females*

|  | **Alcohol consumption in g/week** | | | | | | | | | | |
| --- | --- | --- | --- | --- | --- | --- | --- | --- | --- | --- | --- |
| **Age** | *Analysed sample* (N = 990)* | | *Females* | | *Females above cutoff*** | | *Males* | | *Males above cutoff**** | | *p (mean f vs. m)* |
|  | Mean | N | Mean | N | % | N | Mean | N | % | N |  |
| 14 | 0.73 | 124 | 0.16 | 69 | 1.1 | 1 | 1.43 | 55 | 0.0 | 0 | 0.86 |
| 15 | 10.5 | 162 | 2.25 | 91 | 0.0 | 0 | 21.2 | 71 | 2.8 | 2 | < 0.01 |
| 16 | 23.8 | 138 | 14.7 | 78 | 7.7 | 6 | 35.6 | 60 | 8.3 | 5 | < 0.01 |
| 17 | 36.5 | 110 | 31.1 | 67 | 16.5 | 11 | 44.9 | 43 | 11.6 | 5 | 0.28 |
| 18 | 44.5 | 129 | 32.4 | 77 | 14.3 | 11 | 62.4 | 52 | 13.5 | 7 | 0.018 |
| 19 | 59.9 | 109 | 48.7 | 72 | 25.0 | 18 | 81.9 | 37 | 16.2 | 6 | 0.038 |
| 20 | 59.5 | 115 | 47.6 | 71 | 19.7 | 14 | 78.7 | 44 | 13.6 | 6 | 0.025 |
| 21 | 72.0 | 103 | 57.1 | 65 | 29.2 | 19 | 97.5 | 38 | 31.6 | 12 | 0.027 |

General analysed samples (dropping missing cortisol and TICS data and unplausible cortisol data (see methods)). Data are unweighted. **10g per day, ***20g per day (Burger et al. 2004)

Burger, M., Brönstrup A., & Pietrzik K. (2004). Derivation of tolerable upper alcohol intake levels in Germany: a systematic review of risks and benefits of moderate alcohol consumption. *Preventive Medicine*, 39 (1), 111-127.
